# Cryptococcal antigen titers and semi-quantitative assay scores among people with HIV-associated cryptococcal antigenemia

**DOI:** 10.1128/jcm.00886-25

**Published:** 2026-01-15

**Authors:** Tshiama M. Mwamba, Nozuko P. Blasich, Lindi M. Coetzee, Rudzani Mashau, Nelesh P. Govender

**Affiliations:** 1 Wits Mycology Division, School of Pathology, Faculty of Health Sciences, University of the Witwatersrand 37708 , Johannesburg, South Africa; 2 Centre for Healthcare-Associated Infections, Antimicrobial Resistance and Mycoses, National Institute for Communicable Diseases, a Division of the National Health Laboratory Service 70687, Johannesburg, South Africa; 3 National Priority Programmes CD4 Unit, National Health Laboratory Service 70685, Johannesburg, South Africa; 4 Division of Medical Microbiology, University of Cape Town 37716, Rondebosch, South Africa; 5 Institute of Infection and Immunity, City St George’s University of London 4895, London, United Kingdom; 6 MRC Centre for Medical Mycology, University of Exeter 601337, Exeter, United Kingdom; University of Utah, Salt Lake City, Utah, USA

**Keywords:** *Cryptococcus*, cryptococcal antigenemia, cryptococcal antigen, lateral flow assay, semi-quantitative, cryptococcal meningitis, South Africa

## Abstract

While South African guidelines recommend a lumbar puncture (LP) to exclude cryptococcal meningitis (CM) among all people with a newly positive cryptococcal antigen (CrAg) test, irrespective of CM symptoms, this is not always feasible. High blood CrAg lateral flow assay (LFA) titers are associated with concurrent CM and increased mortality. Single-strip CrAg semi-quantitative (SQ) tests could risk-stratify people with antigenemia. Consecutive fresh LFA-positive remnant plasma samples from a CD4 laboratory network collected between April and July 2021 were retested. We described LFA titers, CrAgSQ scores, and the proportion with cerebrospinal fluid (CSF) collected 28 days before or after a positive CrAg screening test. Of 2,240 re-tested plasma samples from unique patients, 2,166 (97%) were confirmed LFA-positive. The median LFA titer was 640 (IQR, 40–5,120), 63% (1,354/2,166) had a titer of ≥160, and 52% (1,124/2,166) had SQ scores of ≥3+. Only 31% (662/2,166) had a CSF sample collected 28 days before or after a CrAg LFA-positive test; 60% (398/662) had confirmed CM. More than half of the people with cryptococcal antigenemia had a blood CrAg titer of ≥160 or a CrAgSQ score of ≥3+, both previously shown to confer a high risk of concurrent CM. Of 3 in 10 who had an LP, most had CM, suggesting that meningitis symptoms prompted LP. Healthcare worker support/training is required to improve adherence to the universal LP recommendation. When immediate LP is not feasible, blood CrAgSQ testing can rapidly identify people at the highest risk of CM who require urgent referral for LP.

**IMPORTANCE:**

A majority of patients with HIV-associated cryptococcal antigenemia identified through a large screening program in South Africa had high cryptococcal antigen titers and thus an elevated risk of concurrent meningitis and death. Despite this, a relatively small proportion had a lumbar puncture to definitively exclude meningitis. Routine CrAg semi-quantification can help to stratify patients at higher risk for meningitis and guide clinicians’ management, but performing a full range of titers for all CrAg-positive blood samples increases costs and is labor-intensive. An alternative approach is to use a single test strip, which yields a semi-quantitative score.

## INTRODUCTION

Cryptococcal antigen (CrAg) screening is primarily intended to identify early cryptococcal disease among people living with advanced HIV disease before progression to cryptococcal meningitis (CM); however, some patients with a CrAg-positive screening test may already have concurrent subclinical or symptomatic CM (1, 2). Wake et al. (3) found that approximately one-third of people with HIV-associated antigenemia had concurrent subclinical CM, with a blood CrAg lateral flow assay (LFA) titer cut-off of >160 predicting CM. However, the titer cut-off to predict CM or death varies by the test method, study design, and study population; a pooled summary of four cohorts from different countries reported an LFA titer threshold of ≥160 (2, 4–8). In Botswana, where ART-experienced patients with antigenemia had a lower risk of death than those who were ART-naïve (9), an LFA titer of >40 was associated with 6-month mortality. Jarvis et al. (10) reported an association between increasing CrAgSQ assay scores generated by a single-strip semi-quantitative (SQ) assay and concurrent CM/10-week mortality.

A lumbar puncture (LP) is strongly recommended to exclude subclinical CM among people with antigenemia (1). However, patients without meningitis symptoms may opt not to accept an LP (1, 3), or patients who screen CrAg-positive at primary healthcare facilities may not be appropriately referred to a facility where an LP can be performed. Adherence to this LP recommendation in the South African reflex CrAg screening program is not well documented. This is owing, in part, to the difficulties of following up CrAg-positive patients in a health system without unique patient identifiers and lacking a unified electronic health information system.

All CD4 laboratories in South Africa reflexively perform a qualitative CrAg LFA on remnant blood from patients with a CD4 count of <100 cells/µL (11). Although semi-quantification of CrAg levels by titer cannot definitively confirm or exclude meningitis, this may provide useful information to clinicians for cases in which an LP is not available or a patient declines consent for LP. However, performing a full range of titers for all CrAg-positive samples increases costs and is labor-intensive, especially in laboratories that test large numbers of samples. One option could be to adopt a recommendation (12) to test two sample dilutions (i.e., 1:160 and 1:1280 to stratify patients into three titer risk groups [low: <160, medium: ≥160], and high: ≥1,280). Another option would be to use a CrAgSQ assay (Immy, Norman, OK), which provides semi-quantitative results using a single test strip and has a high sensitivity and specificity comparable to the conventional LFA (10, 13). In a Ugandan study, the CrAgSQ assay was validated for use on CSF from 87 participants and found to have 100% sensitivity and specificity in patients with HIV-associated CM (14).

We aimed to determine LFA titers and CrAgSQ test scores on LFA-positive plasma samples from a large sample of people with CD4 counts of <100 cells/µL tested in South Africa’s reflex screening program and the proportion of LFA-positive individuals who had evidence of an LP in routine care.

## MATERIALS AND METHODS

### Study population and sample collection

We performed a laboratory-based cross-sectional study in which consecutive CrAg LFA-positive remnant blood samples in ethylenediamine-tetraacetic acid (EDTA) tubes were shipped to a reference laboratory at the National Institute for Communicable Diseases (NICD) for re-testing using the same LFA method (Immy, Norman, Oklahoma, USA). These consecutive settled plasma samples, which were collected during a 4-month period (1 April to 31 July 2021) from patients with a CD4 count of <100 cells/µL, had initially tested CrAg LFA-positive by National Health Laboratory Service (NHLS) CD4 laboratories in the routine screening program. Communication was sent out to all NHLS laboratories informing them of the study to send all remnant EDTA samples that were CrAg LFA-positive.

### Sample processing

**Fig 1:**
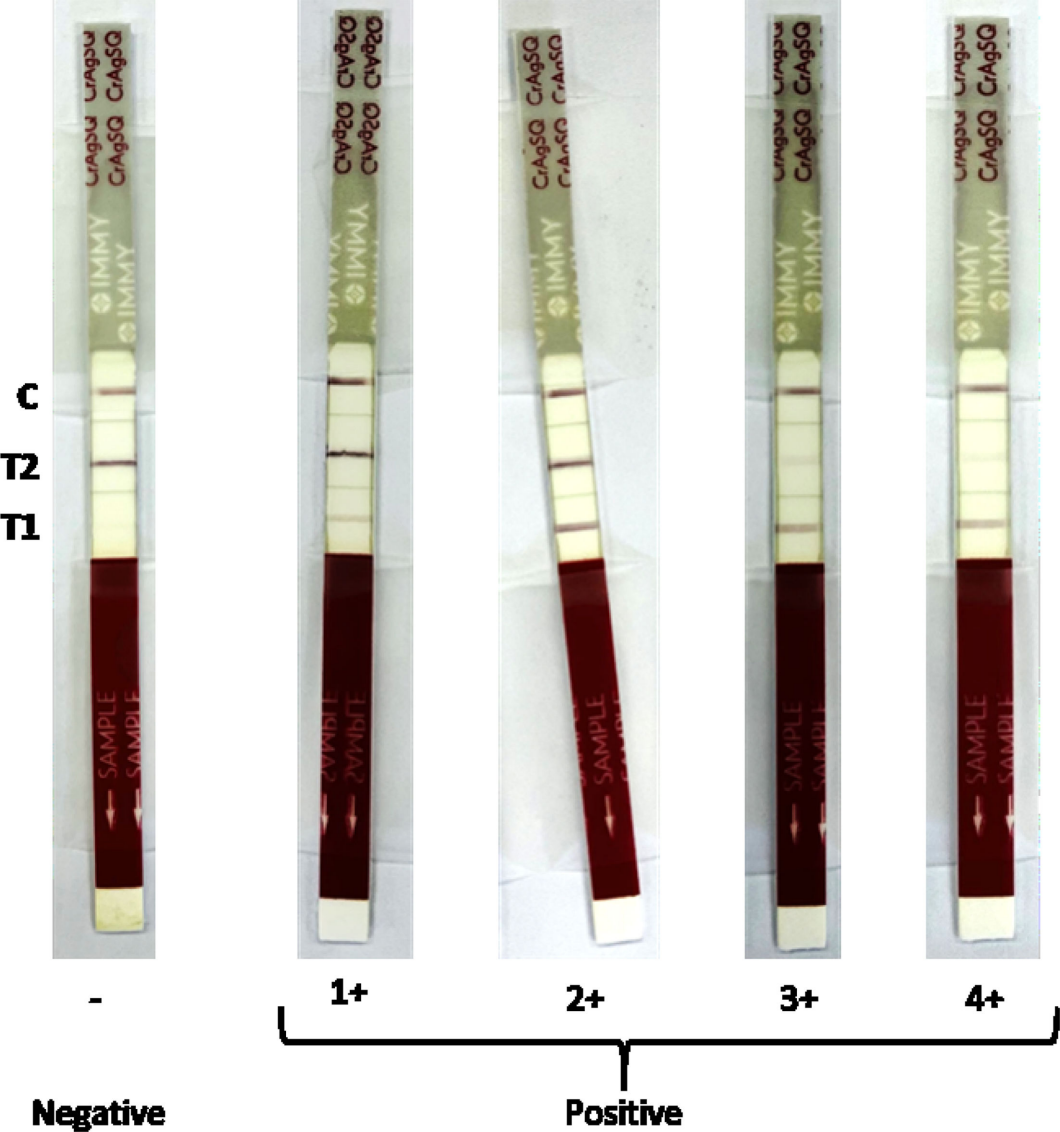
Visual representation of the CrAgSQ results. The picture shows examples from the results obtained in this study. The C line should always be present for a valid result. The CrAgSQ scoring is based on line intensity patterns of the T1 and T2 lines as follows; positive results if: T1 < T2  = 1+; T1 = T2  = 2+; T1 > T2  = 3+; only T1 =  4+; only C =  5+; and negative results if only T2 and C lines are present. A 5+ was not obtained for any of the samples in our study.

The conventional CrAg LFA and CrAgSQ assays were both performed as per the manufacturer’s instructions on settled plasma samples by two trained independent readers who were blinded to the routine results and to each other’s reads. The conventional LFA provides a qualitative CrAg result within 10 minutes, excluding preparation time. Semi-quantitative results can be obtained by performing serial dilutions of the original sample to obtain titers. CrAg titers were determined using 10 dilutions of each sample (from 1:5 to ≥1:2,560). Titers recorded at a dilution of 1:2,560 were recorded as ≥2,560 since dilutions stopped at this point, and there was no further sample dilution to confirm that this would be the last positive titer. Readers 1 and 2 titer results were considered concordant if they were within 2 dilutions of each other. The CrAgSQ assay uses a scoring system to indicate increasing CrAg quantities in a sample and also provides results within 10 minutes, excluding preparation time. The CrAgSQ strip has three possible lines: test 1 (T1), test 2 (T2), and control (C), and the scoring is based on line intensity patterns of the T1 and T2 lines (Fig. 1). Readers 1 and 2 score results were considered concordant if there was only one score difference in either direction.

### Retrospective CM laboratory data

Participant identifiers (e.g., laboratory episode number, hospital folder number, name, date of birth) for each CrAg-positive sample were used to retrieve information on cerebrospinal fluid (CSF) results from the NHLS TrakCare laboratory information system and/or the NICD’s Surveillance Data Warehouse. We searched for CSF specimens collected within 28 days before or after a positive reflex CrAg LFA on plasma. Cryptococcal meningitis was confirmed on CSF by fungal culture or positive India ink microscopy, or a positive CSF CrAg LFA. We defined prior CM as an episode diagnosed before the day of collection of the positive reflex plasma CrAg LFA, and concurrent CM as an episode diagnosed on the same day or 28 days after a positive reflex plasma CrAg LFA. We also searched for episodes of CM diagnosed using CSF specimens collected >28 days after a positive reflex CrAg LFA on plasma.

### Data analysis

The concordance between reflex CrAg LFA results reported by NHLS CD4 laboratories and CrAg LFA results at NICD was described. We also calculated the sensitivity of the CrAgSQ assay compared to the qualitative CrAg LFA on plasma as the reference method (NICD). We assessed inter-rater reliability for the CrAg LFA and CrAgSQ assays using all samples and used Cohen’s Kappa statistic to measure the agreement between the CrAgSQ assay (index) versus CrAg LFA titers (15). The inter-rater reliability and inter-reader percent agreement for titers and SQ scores were also assessed (reader 1 versus reader 2) using the Kappa statistic. Since the CrAgSQ test has ordered categorical values, we calculated a second weighted Kappa with 80% wt given to results that were within one category between the two reviewers (e.g., readings of 3+ and 4+); 60% wt given to results within two categories; 40% wt for results within three categories; and 20% wt for a discrepancy of four categories; and 0% wt for a discrepancy of five categories. The CrAg LFA titer results also comprised ordered categorical values, and a weighted Kappa was calculated for LFA titers with 11 categories. The association between CrAgSQ scores and CrAg titer values was evaluated and displayed graphically. The proportion of CrAg-screened individuals with an LP and with concurrent CM was calculated by plasma LFA titer and CrAgSQ category. All analyses were performed using STATA version 15.1 (Stata Corporation, College Station, TX).

## RESULTS

**Fig 2:**
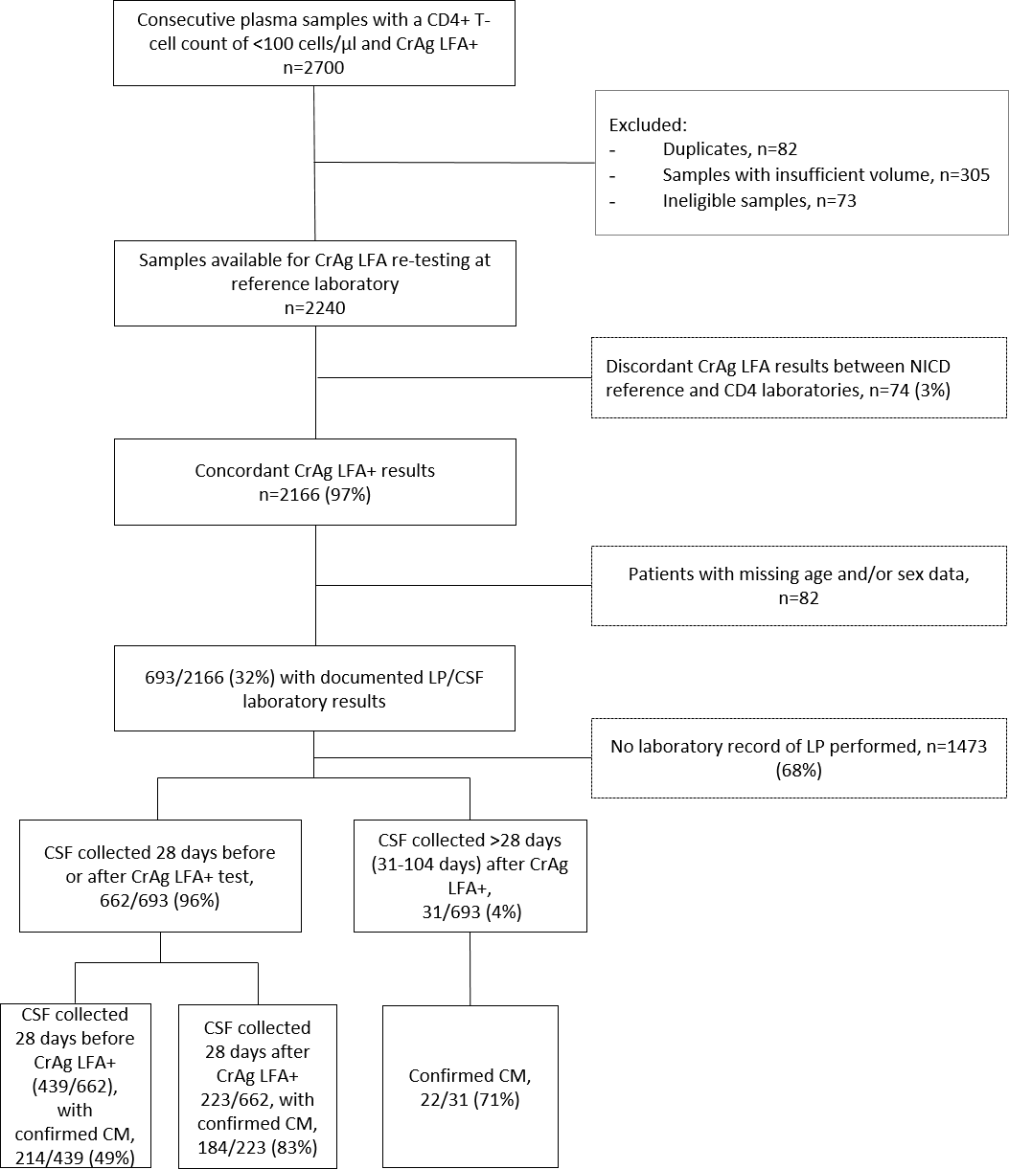
Flow chart of 2,700 patients with a plasma CrAg LFA-positive test and a CD4 T-cell count of <100 cells/µL identified through the reflex CrAg screening program in South Africa, 1 April–31 July 2021. Ineligible samples mainly comprised of hemolysed samples that could not be retested as that would prevent accurate interpretation of results.

A total of 2,700 fresh LFA-positive plasma samples were received from 45 of 47 NHLS CD4 laboratories nationwide for re-testing at NICD. After excluding duplicate samples (*n* = 82), samples with insufficient volumes for re-testing (*n* = 305), and ineligible samples (*n* = 73) (of which five were collected outside the defined study period, 53 were hemolysed samples that could not be re-tested, and 15 were LFA-negative samples sent in error from CD4 labs), a total of 2,240 unique samples were available (Fig. 2). Of these, 2,166 (97%) had LFA-positive results at NICD and were thus concordant with LFA results obtained at NHLS CD4 laboratories. Laboratory LP/CSF records were matched for 693/2,166 patients (32%). Of these, 439 CSF samples had been collected in the 28 days before a positive CrAg LFA, 223 had been collected within 28 days after a positive CrAg LFA, and the remaining 31 were collected after 28 days. The proportions with confirmed CM in each group are reported in Fig. 2.

### Characteristics of 2166 participants with concordant LFA-positive results

Among those with concordant LFA-positive results, their median age was 38 years (interquartile range [IQR], 32–45 years; 2,097 with available data), and 57% (1,227/2,153) were male (Table 1). The median CD4 count was 31 cells/µL (IQR, 13–56 cells/µL), and 41% (887/2,166) had a CD4 count of <25 cells/µL. A large proportion of participants (43%, 940/2,166) had LFA titers of ≥2560, with an overall median LFA titer of 640 (IQR, 40–5,120). More than half (63%, 1,354/2,166) had an LFA titer of ≥160. Males had higher median LFA titers (640; IQR, 40–5,120) than females (160; IQR, 20–5,120) (*P* < 0.001). About one-third of the participants had CrAgSQ scores of either 1+ or 3+, while smaller proportions had scores of 2+ or 4+. More than half had a CrAgSQ score of ≥3+ (52%, 1,124/2,166).

**TABLE 1:** Baseline characteristics of participants with concordant LFA-positive results at CD4 laboratories versus NICD reference laboratory, *N* = 2166 (74 excluded with discordant LFA results)^*b*^

Characteristics	Total *N* with data	Value (median, IQR) or (*n*,%)
Median age in years (IQR)	2,097	38 (32–45)
Male sex	2,153	1,227 (57)
Median CD4 count (cells/µL) (IQR)		31 (13–56)
CD4 category (cells/µL)	2,166	
0–24		887 (41)
25–49		611 (28)
50–74		409 (19)
75–99		259 (12)
Median plasma CrAg LFA titer (IQR)	2,166	640 (40–5,120)
Plasma CrAg LFA titer category	2,166	
1		90 (4.2)
5		75 (3.5)
10		152 (7.0)
20		176 (8.1)
40		171 (7.9)
80		148 (6.8)
160		148 (6.8)
320		110 (5.1)
640		71 (3.3)
1,280		85 (3.9)
≥2,560		940 (43.4)
Median CrAgSQ score (IQR)	2,140	3+ (1+ to 3+)
CrAg SQ score category	2,140	
Negative^*a*^		26 (1.2)
1+		771 (35.6)
2+		245 (11.3)
3+		769 (35.5)
4+		355 (16.4)
Province	2,166	
KwaZulu-Natal		561 (26)
Gauteng		422 (19)
Eastern Cape		359 (17)
Western Cape		302 (14)
Limpopo		158 (7)
Mpumalanga		131 (6)
North West		127 (6)
Free State		99 (5)
Northern Cape		7 (<1)

^*a*^ Negative for CrAgSQ score category: these patients had positive CrAg LFA results, with tiers ≤1:20.

^*b*^ CrAg LFA: Cryptococcal antigen lateral flow assay; NICD: National Institute for Communicable Diseases.

### CrAgSQ versus CrAg LFA

**Fig 3:**
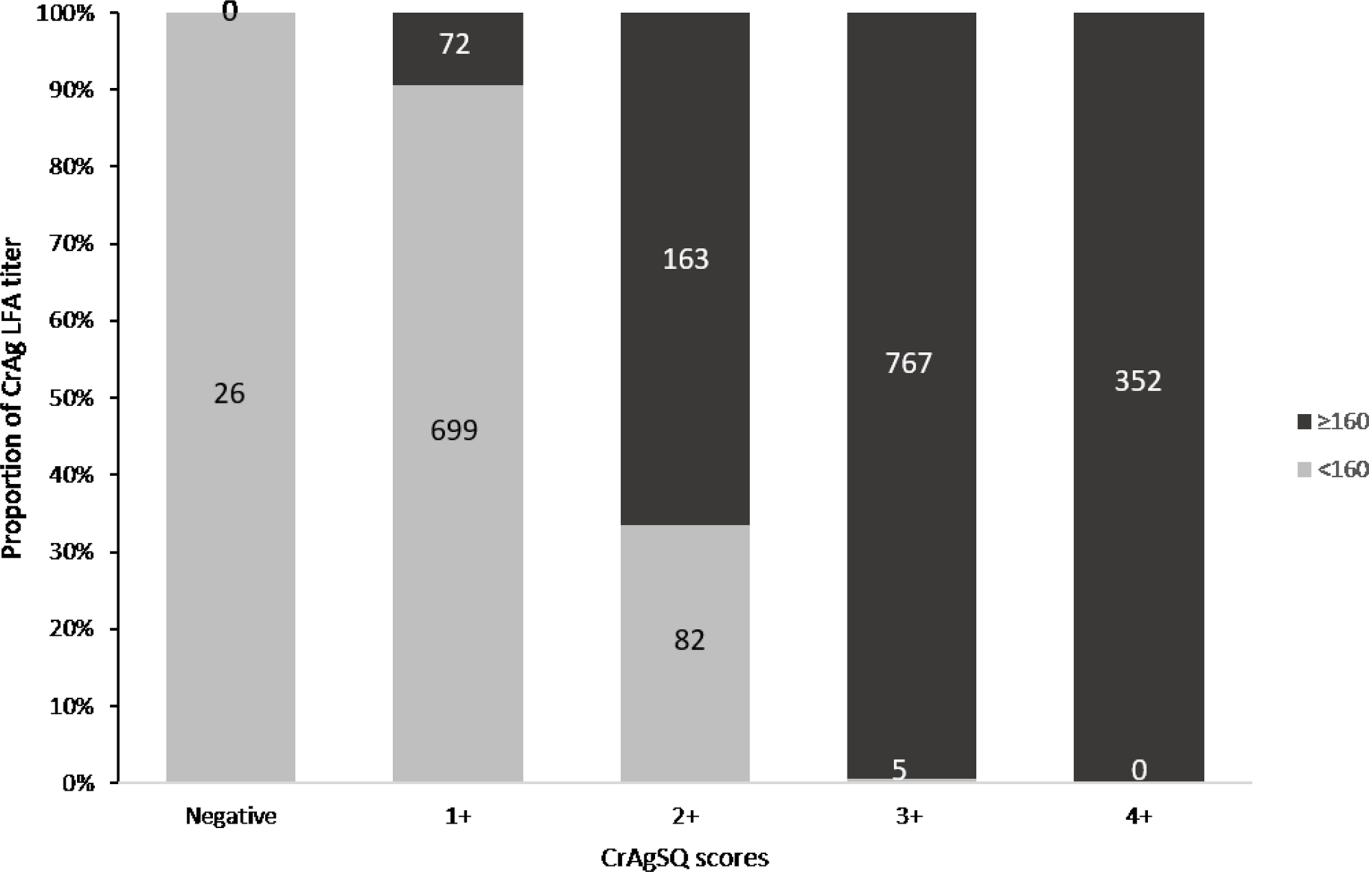
Proportion of CrAg LFA titers above and below the 160 threshold within each CrAgSQ category, in patients with concordant LFA-positive results, *n* = 2,166. CrAgSQ: cryptococcal antigen semi-quantitative, LFA: lateral flow assay.

Compared to the NICD CrAg LFA results as a reference, the CrAgSQ assay was 98.8% sensitive (95% CI, 98.2%–99.2%). There were 26 LFA-positive/CrAgSQ-negative samples, all of which had LFA titers of ≤20 (17 with a titer of 1, six with a titer of 5, two with a titer of 10, and one with a titer of 20). Of the 74 LFA-negative samples at NICD, the CrAgSQ assay was also negative for 69 (93.2%), while the other 5 samples were CrAgSQ-positive (all with a score of 1+). We compared CrAgSQ scores with LFA titers among 2,166 patients with concordant LFA-positive results. CrAgSQ scores ranged from 1+ to 4+ and LFA titers ranged from 1 to ≥2,560. Of 812 participants (37% of total) with an LFA titer of <160, 86% (*n* = 699) had a CrAgSQ score of 1+, followed by 10% (*n* = 82) with CrAgSQ score of 2+ and 1% (*n* = 5) with a score of 3+ (Fig. 3). Only 3% (*n* = 26) had negative CrAgSQ results, and these corresponded to LFA titers of ≤20. Of the 1354 (63% of total) with LFA titers ≥160, 5% (*n* = 72) had a 1+ score, 12% (*n* = 163) had CrAgSQ score of 2+, 57% (*n* = 767) had a CrAgSQ score of 3+, 26% (*n* = 352) had a CrAgSQ score of 4+, and none had a negative result or a 5+ score (Fig. 3). For the CrAgSQ scores, the readers had identical results for 84% of the samples, and 97% were within ±1 CrAgSQ score (Cohen’s Kappa 0.87, *P* < 0.001) (Table 2). For CrAg LFA titers, the readers had identical results for 71% of the samples, and 97% of the titer results were within ±1 titer for each reader (Cohen’s Kappa 0.90, *P* < 0.001).

**TABLE 2:** Inter-rater reliability of the CrAgSQ assay in EDTA-blood samples from all patients with available samples for re-testing, *N* = 2,240

A. Inter-rater agreement								
		Reader 2						
		0	1+	2+	3+	4+	5+	Total
Reader 1	**0**	92	3	0	0	0	0	95
	**1+**	10	711	48	7	0	0	776
	**2+**	0	26	177	40	2	0	245
	**3+**	1	6	28	638	94	2	769
	**4+**	0	4	0	88	261	2	355
	**5+**	0	0	0	0	0	0	0
	**Total**	103	750	253	773	357	4	**2,240**

B. Inter-rater reliability (Cohen’s Kappa Statistic)					
Inter-rater reliability	% agreement		Cohen’s Kappa	SE	*P* value
	Expected	Observed			
Unweighted	27.4	83.88	0.778	0.012	<0.0001
Weighted^a^	73.43	96.54	0.8696	0.016	<0.0001

^a^ To account for the ordered categorical data and assess the degree of disagreement, disagreements were weighted in a linear way; with six categories, cases in adjacent categories were weighted by factor 0.8, those with a distance of two categories weighted 0.6, those with a distance of three categories weighted 0.4, those with a distance of four categories weighted 0.2, and those with a distance of five categories weighted 0.

### Prior and concurrent CM

**Fig 4:**
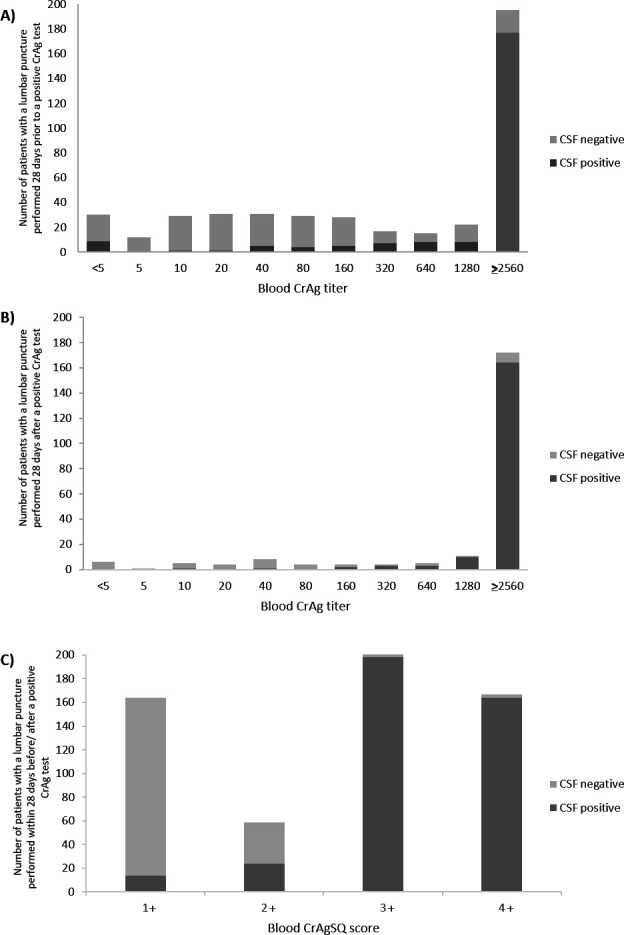
Total number of patients with cryptococcal antigenemia who had CSF available for analysis, and the proportion with confirmed CM (i.e., CSF cryptococcal test positive) by (**A**) CrAg titer, among patients who had an LP performed 28 days before a CrAg LFA-positive test; (**B**) CrAg titer, among patients who had an LP performed within 28 days after a CrAg LFA-positive test. (**C**) CrAgSQ score, among patients who had an LP performed 28 days either before or after a CrAg LFA-positive test. Note that CSF results were only available for 31% of all patients, and thus this sample is probably highly selected for CM.

Only 31% (662/2,166) of patients with antigenemia had a record of CSF collected 28 days before or after a positive blood CrAg LFA. Of 662 patients with an LP performed, 398 (60%) had confirmed CM. Of these 662, 439 individual CSF samples were collected 28 days before a positive CrAg LFA test, and 49% (214/439) had confirmed prior CM. Of 223 patients who had CSF samples collected within 28 days after their positive CrAg LFA (only 10% of total), 83% (184/223) had confirmed concurrent CM. Among those with concurrent CM, their median age was 37 years (IQR, 32–44 years), and 58% (107/184) were male. Their median CD4 count was 26 cells/µL (IQR, 11–46 cells/µL). Ninety-eight percent () had an LFA titer of ≥160, and 175 had a CrAgSQ score of ≥3+. Thirty-one participants had CSF collected >28 days (range, 31–104 days) after their positive CrAg LFA, and of these, 22 had CM (71%) (Fig. 2). The proportion of patients with CrAg titer ≥160 who had CM (471/662, 71%) was higher than those with a CrAg titer <160 (191/662, 29%), *P* < 0.001 (Fig. 4A and B). Similarly, the proportion of patients with a CrAgSQ score of ≥3+ (420/662, 63%) who had CM was higher than those with a CrAgSQ score of ≤2+ (242/662, 37%), *P* < 0.001 (Fig. 4C).

## DISCUSSION

We described more than 2,000 people with HIV-associated cryptococcal antigenemia who were identified through a national screening program in South Africa. More than half of the patients with antigenemia had a blood CrAg titer of ≥160 or a CrAgSQ score of ≥3+. CrAgSQ scores of 3+ and 4+ generally correlated with LFA titers of ≥160. Other studies have shown that titers of ≥160 and CrAgSQ scores of ≥3+ were associated with subclinical CM and mortality, though these cut-offs vary by study (3, 8, 12, 16, 17). However, despite these findings, 31% of patients with cryptococcal antigenemia in our study had CSF collected 28 days before or after their positive CrAg test. Only 10% had CSF collected within 28 days after their positive screen. Sixty percent of the patients who had an LP had laboratory-confirmed CM, and thus we speculate that they must have had overt symptoms of meningitis which prompted the LP.

A “screening” LP is strongly recommended to be performed following a positive blood CrAg test, regardless of meningitis symptoms, to exclude CM. However, only a minority with antigenemia in our study had CSF submitted for laboratory analysis. This could be explained by asymptomatic patients declining LP (18, 19) or by LP not being offered by healthcare workers (20, 21). In a recent Zambian study, a completed LP was associated with increased patient knowledge (20). The high proportion of CM among those who had an LP suggests that meningitis symptoms were the main trigger for an LP. Training healthcare workers to perform a screening LP even among patients without meningitis symptoms, making consumables for LP readily available, simplifying health system referral pathways to facilitate screening LPs, and enhancing patient education may improve LP uptake (20). An alternative approach to identify patients at high risk of CM is semi-quantification of CrAg in blood, either through LFA titers or a CrAgSQ assay.

Although performing CrAg titers and reading the results is simple, this is also labor-intensive, and the test cost increases with each dilution. For example, if the antigenemia prevalence is 5% and 10 dilutions are performed on every LFA-positive sample, 150 test strips would be needed for every 100 screened patients, a 50% increase in cost. Because reflex CrAg screening is performed at high volumes in South Africa’s CD4 laboratories, a single-step CrAgSQ assay is an attractive tool. Currently, the price of the CrAgSQ test is approximately double that of a CrAg LFA in South Africa, and the cost of a CrAgSQ test for one patient may thus work out to be 5× cheaper than an LFA if titers were performed in 10 dilutions. However, the cost-effectiveness of replacing the LFA with the CrAgSQ test in a screening program is not yet established. Turnaround times increase when performing CrAg LFA titers instead of testing blood with a single CrAgSQ assay strip. The interpretation of a CrAgSQ reading on the other hand is also complex. The test uses a three-line system on a wicking strip and results in negative, 1+, 2+, 3+, 4+, or 5+ scores. The interpretation is based on the arrangement of test 1 (T1; sandwich formation), test 2 (T2; competitive inhibition), and control (C) lines, with T1 being the first line encountered by the wicking process, followed by a T2 line and C being the last (14). Depending on the combination of lines formed and their intensities, CrAg SQ scores are assigned. In our study, CrAgSQ score interpretation was performed in real time by two experienced laboratory scientists using the interpretation card provided with the kit. The study samples were re-tested over a period of 6 months, and we found that reading CrAgSQ results became easier with increasing bench time, with less dependence on the interpretation card.

In addition to the complex interpretation of results, Skipper et al. (13) argued that inter-reader variability in reading CrAgSQ scores might also complicate the assay. However, inter-reader variability already exists in the interpretation of the conventional LFA and titer test results. While the inter-reader variability in reading CrAgSQ scores was low, that is, high agreement, in research studies (≥99% agreement in Jarvis et al. [10] and Skipper et al. [13] versus 97% agreement in our study), this issue might be more pertinent in routine settings with less-experienced readers. The introduction of such an assay into a routine service would require additional staff training. Cube readers or artificial intelligence-reading assistance could be explored to improve accuracy, consistency, and access to results, but may be limited by cost, equipment needs, and risks of over-reliance on this technology or misclassification. Ongoing treatment trials for cryptococcal antigenemia (e.g. ISRCTN30579828) will provide clearer answers to the utility and cost-effectiveness of this CrAgSQ assay for risk stratification and enhanced antifungal treatment.

A limitation of this study is selection bias; patients who received an LP were probably those with overt meningitis symptoms, limiting the generalizability of findings to all patients with antigenemia, but at the same time highlighting the lack of adherence to the universal LP recommendation. Another limitation is that the CrAgSQ assay requires reader training, and although inter-reader agreement was high in this and other research settings, variability may be greater in routine practice with less-experienced staff. No follow-up data in this cross-sectional study meant that outcomes and the predictive value of high titers on meningitis or all-cause mortality could not be assessed. We performed this study in a country with a high national HIV prevalence, a large population with advanced HIV disease, and a relatively high prevalence of cryptococcal disease. Our recommendations may not be generalizable to lower-prevalence settings.

In conclusion, a large proportion of patients with antigenemia identified through routine screening had high CrAg levels, yet very few had an LP. A very large proportion of those with an LP had confirmed CM, suggesting that meningitis symptoms remained the main trigger for an LP. We recommend regular and enhanced healthcare worker training and patient health literacy to improve uptake of screening LPs for all those with antigenemia. In settings where an LP is not feasible, not immediately available, or is declined by patients, stratifying patient risk using blood LFA titers or a single-strip CrAgSQ test can identify people at the highest risk of CM for urgent LP referral. Nevertheless, further evaluation in prospective treatment trials of antigenemia is required.

## Data Availability

The data sets used and/or analyzed during the current study are available from the corresponding author on reasonable request.
